# Microembolic signals in transcranial Doppler and the functional outcome of patients with acute ischemic stroke: a meta-analysis

**DOI:** 10.3389/fneur.2026.1911457

**Published:** 2026-09-10

**Authors:** Jun Qiao, Zhikun Zhang, Yujin Feng, Yi Wang

**Affiliations:** 1 Department of Psychiatry, The First Hospital of Hebei Medical University, Shijiazhuang, China; 2 Department of Radiology, The First Hospital of Hebei Medical University, Shijiazhuang, China; 3 Department of Ultrasound, The Second Hospital of Hebei Medical University, Shijiazhuang, China

**Keywords:** functional outcome, ischemic stroke, meta-analysis, microembolic signals, transcranial Doppler

## Abstract

**Background:**

Microembolic signals (MES) detected by transcranial Doppler ultrasonography (TCD) reflect ongoing cerebral embolization and may serve as a marker of stroke instability. However, the association between MES and functional outcomes in patients with acute ischemic stroke (AIS) remains uncertain. This meta-analysis aimed to evaluate the prognostic significance of MES for poor functional outcomes after AIS.

**Methods:**

PubMed, Embase, Web of Science, Wanfang Data, and CNKI databases were searched for cohort studies comparing functional outcomes between AIS patients with and without MES. The primary outcome was poor functional outcome, defined as a modified Rankin Scale score of 3–6 during follow-up. Odds ratios (ORs) and 95% confidence intervals (CIs) were pooled using a random-effects model accounting for the influence of potential heterogeneity.

**Results:**

Thirteen cohort studies involving 1,777 patients with AIS were included, of whom 361 (20.3%) had detectable MES. Overall, MES was associated with a significantly increased risk of poor functional outcome (OR: 2.85, 95% CI: 2.16–3.75; *p* < 0.001) with no significant heterogeneity (*I*^2^ = 0%). Subgroup analyses demonstrated consistent results across prospective and retrospective studies, and the observed association was not significantly influenced by patient age, sex distribution, endovascular treatment status, TCD monitoring duration, analytical model, or study quality (all *p* for subgroup difference > 0.05). MES was associated with poor functional outcomes at discharge (OR: 3.60), 14 days (OR: 2.69), and 90 days (OR: 3.15).

**Conclusions:**

MES detected by TCD are associated with an increased risk of poor functional outcome in patients with AIS. However, their independent and incremental prognostic value beyond established clinical and imaging predictors requires further confirmation.

**Systematic Review Registration:**

The review protocol was prospectively registered in PROSPERO (CRD420261425262).

## Introduction

Acute ischemic stroke (AIS) is one of the leading causes of death and long-term disability worldwide, imposing a substantial burden on patients, caregivers, healthcare systems, and society (1, 2). Despite advances in reperfusion therapies, secondary prevention, and stroke unit care, a considerable proportion of patients experience persistent neurological deficits and functional dependence after stroke (3, 4). Poor functional outcomes are associated with reduced quality of life, increased healthcare utilization, loss of productivity, and higher long-term mortality (5). Therefore, early identification of patients at increased risk of poor functional outcome is essential for optimizing risk stratification, guiding clinical decision-making, and improving individualized management strategies (6). Although several clinical, imaging, and laboratory predictors have been proposed, additional prognostic markers that can be readily incorporated into routine clinical practice remain needed (7, 8).

Microembolic signals (MES) are transient high-intensity signals detected within intracranial arteries and are considered surrogate markers of ongoing cerebral embolization (9, 10). They are typically identified using transcranial Doppler ultrasonography (TCD), a noninvasive, bedside, real-time, and relatively inexpensive technique that allows continuous monitoring of cerebral blood flow and embolic activity (9, 10). MES may represent platelet aggregates, thrombotic fragments, or atherosclerotic debris originating from unstable vascular or cardiac sources (11). From a pathophysiological perspective, the presence of MES may indicate persistent embolic activity, unstable atherosclerotic plaques, inadequate thrombus resolution, or ongoing cardioembolic phenomena, all of which may contribute to recurrent cerebral ischemia, enlargement of infarct volume, impaired reperfusion, and delayed neurological recovery (12–14). Accordingly, MES have been proposed as a potential marker of disease instability and poor prognosis after AIS (15).

A growing number of observational studies have evaluated the prognostic significance of MES in patients with AIS (9). However, the results have been inconsistent. Some studies reported that MES were associated with poor functional outcomes (14, 16–21), whereas others failed to demonstrate a significant relationship (22–27). Previous systematic reviews primarily focused on the prevalence of MES and their association with recurrent cerebrovascular events (28, 29). In a previous meta-analysis published in 2021, Sudheer et al. included 58 studies and demonstrated that MES were associated with an increased risk of recurrent cerebral ischemia but not with poor functional outcomes (28). However, the analysis of functional outcomes was based on only three studies involving 443 patients, resulting in wide confidence intervals and limited statistical power (28). Moreover, several relevant cohort studies evaluating MES and functional outcomes have subsequently become available (14, 19–21, 26, 27). Therefore, we performed an updated systematic review and meta-analysis to comprehensively evaluate the association between MES detected by TCD and poor functional outcomes in patients with AIS.

## Methods

This meta-analysis was conducted in accordance with recognized standards for systematic reviews and meta-analyses. The methodology adhered to the recommendations of the PRISMA 2020 guideline (30) and the Cochrane Handbook for Systematic Reviews of Interventions (31), covering all stages from protocol development and literature screening to data extraction, quantitative synthesis, and interpretation of findings. The review protocol was prospectively registered in PROSPERO (CRD420261425262).

### Database search

A comprehensive search of the literature was performed in PubMed, Embase, Web of Science, Wanfang Data, and China National Knowledge Infrastructure (CNKI) to identify all relevant studies, using a combination of the following search terms (1) “microembolic signal^*^” OR “micro embolic signal^*^” OR “embolic signal^*^” OR “high-intensity transient signal^*^” OR “high intensity transient signal^*^” OR “HITS” OR “MES” OR “microembol^*^”; (2) “transcranial Doppler” OR “TCD” OR “transcranial Doppler ultrasonography” OR “transcranial ultrasound” OR “TCCD” OR “transcranial color-coded duplex”; (3) “stroke” OR “ischemic stroke” OR “ischaemic stroke” OR “acute ischemic stroke” OR “cerebral infarction” OR “brain infarction” OR “cerebrovascular disease” OR “cerebrovascular disorder^*^” OR “TIA” OR “transient ischemic attack” OR “transient ischaemic attack”; and (4) “functional outcome” OR “modified Rankin Scale” OR “mRS”. Eligibility was restricted to peer-reviewed full-text studies involving human participants and published in either English or Chinese. To ensure completeness, the reference lists of relevant reviews and included articles were also screened manually for additional records. Database searches covered the period from database inception to April 22, 2026. The complete search strategies used for each database are provided in Supplemental File 1.

### Study inclusion and exclusion criteria

The selection of studies was guided by the PICOS principle:

Population (P): Adult patients (≥ 18 years) with AIS diagnosed clinically and/or radiologically. Studies enrolling mixed stroke populations were eligible if data for AIS patients could be extracted separately.

Intervention/Exposure (I): Presence of MES detected by TCD or transcranial color-coded Doppler (TCCD) during the acute or subacute phase after AIS.

Comparator (C): AIS patients without detectable MES during TCD/TCCD monitoring.

Outcomes (O): The outcome of interest was poor functional outcome, defined as a modified Rankin Scale (mRS) score of 3–6 during follow-up. Studies were required to report adjusted or unadjusted odds ratios (ORs) with 95% confidence intervals (CIs), or provide sufficient data for their calculation.

Study design (S): Prospective or retrospective cohort studies evaluating the association between MES and functional outcome after AIS.

Reviews, meta-analyses, editorials, case reports, animal studies, and non-original articles were excluded. Studies enrolling patients with hemorrhagic stroke, or non-stroke populations were excluded. Studies assessing microembolic lesions detected by imaging rather than TCD/TCCD-detected MES were not eligible. Studies that did not include a MES-negative comparison group, evaluated only MES characteristics among MES-positive patients (e.g., malignant vs. benign MES), reported outcomes other than functional outcome (e.g., recurrence, mortality, cognitive impairment, or neurological progression only), or lacked sufficient data to calculate effect estimates were excluded. For overlapping cohorts, the study with the largest sample size was retained.

### Study quality assessment

All stages of the review process were conducted independently by two investigators, including study identification, eligibility assessment, data extraction, and quality appraisal. Discrepancies were resolved through discussion, with adjudication by a third reviewer when consensus could not be achieved. Study quality was assessed using the Newcastle–Ottawa Scale (NOS) (32), which evaluates the risk of bias in observational studies across participant selection, group comparability, and outcome ascertainment. Scores range from 0 to 9, with a score of 8 or higher indicating high methodological quality.

### Data collection

Two reviewers independently extracted data from eligible studies using a standardized data extraction form developed *a priori*. The extracted information included study characteristics (first author, publication year, country, and study design), patient demographic and clinical characteristics [sample size, mean age, proportion of men, baseline National Institutes of Health Stroke Scale (NIHSS) score, and proportion of patients undergoing endovascular treatment (EVT)], details of the MES assessment protocol (timing of evaluation, monitored vessels, and monitoring duration), the number of patients with detectable MES, follow-up duration, the number of patients with poor functional outcomes during follow-up, and variables included in multivariable-adjusted analyses evaluating the association between MES and poor functional outcomes in patients with AIS.

### Statistical analyses

The association between MES detected by TCD and poor functional outcome of patients with AIS during follow-up was quantified by pooling ORs and corresponding 95% CIs, comparing patients with and without MES on TCD evaluation (31). When available, multivariable-adjusted ORs were preferentially extracted, with estimates from the most comprehensively adjusted model selected when multiple models were reported; otherwise, unadjusted ORs were used. If required, effect sizes and standard errors were calculated from the available 95% CIs or *p* values. Prior to data synthesis, all effect estimates were transformed to the natural logarithmic scale to improve distribution normality and stabilize variances (31). Between-study inconsistency was examined using Cochran's Q statistic and quantified with the *I*^2^ metric (33). Heterogeneity was interpreted as low, moderate, or substantial when *I*^2^ values were < 25%, 25–75%, and > 75%, respectively. Given the anticipated clinical and methodological diversity among studies, pooled estimates were generated using an inverse-variance random-effects model based on the DerSimonian–Laird method (31). The robustness of the findings was investigated through leave-one-out sensitivity analyses, whereby the meta-analysis was repeated after sequential removal of each individual study (34). In addition, prespecified subgroup analyses were conducted according to study design (prospective vs. retrospective), mean patient age, proportion of male participants, EVT status (all patients receiving EVT vs. mixed EVT populations), duration of TCD monitoring for MES detection, follow-up duration, analytical model (univariate vs. multivariable-adjusted), and study quality as assessed by the NOS score. Because the primary analysis aimed to assess the overall association between acute-phase MES and subsequent functional outcome, eligible effect estimates were pooled across follow-up periods, with follow-up duration subsequently examined in subgroup analysis. For all the study-level continuous variables, median values were used as subgroup cutoffs to achieve a balanced distribution of studies between subgroups. Potential small-study effects and publication bias were explored by visual assessment of funnel plots and formally tested using Egger's linear regression method (35). Statistical significance was determined using a two-tailed *p* value of less than 0.05. All quantitative analyses were carried out using RevMan version 5.3 (Cochrane Collaboration, Oxford, UK) and Stata version 17.0 (StataCorp, College Station, TX, USA).

## Results

### Database search results

**Figure 1:**
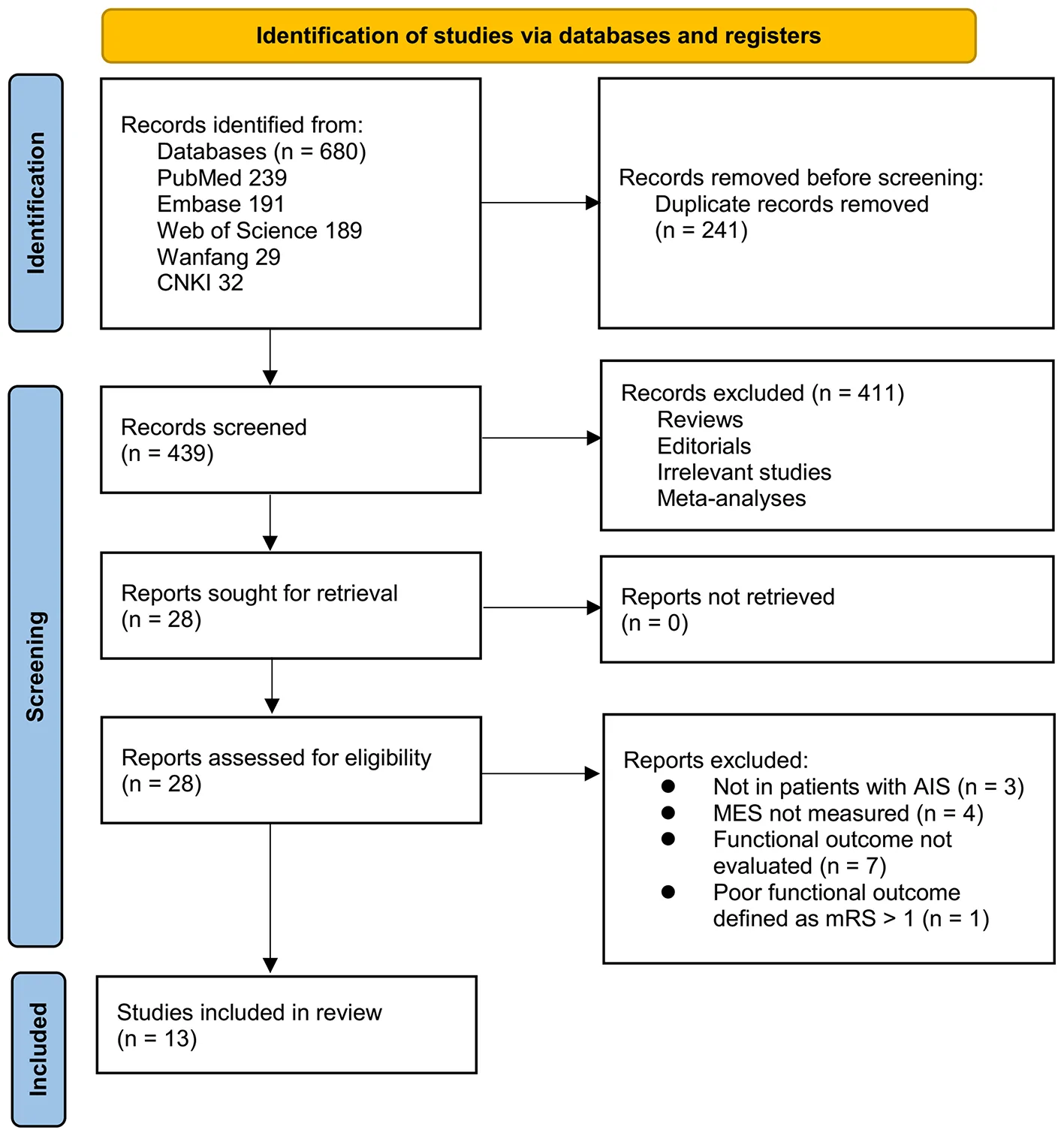
Flow diagram of the study selection process.

Figure 1 summarizes the study identification and selection process. The database search yielded 680 potentially relevant records, of which 241 were duplicates and subsequently removed. Screening of the remaining citations resulted in the exclusion of 411 records at the title and abstract stage. Twenty-eight articles were retrieved for detailed full-text assessment, and 15 were excluded after eligibility evaluation. Ultimately, 13 studies satisfied the inclusion criteria and were included in the meta-analysis (14, 16–27).

### Overview of study characteristics

The main characteristics of the included studies are summarized in Table 1. A total of 13 cohort studies published between 2000 and 2025 were included, comprising 1,777 patients with AIS. Of these, ten studies adopted a prospective cohort design (14, 16, 17, 19, 21–26) and three adopted a retrospective cohort design (18, 20, 27). The studies were conducted predominantly in Europe, including Spain (16), Norway (22), Italy (24), and a multicenter European study involving Portugal, Croatia, Italy, the Czech Republic, and Germany (14), as well as in Asia, including China (17, 19–21, 23, 27). Two studies were conducted in the United States (18, 26), and one study was conducted in Brazil (25), indicating moderate geographic diversity. The mean age of the study populations ranged from 58.2 to 78.0 years, the proportion of men ranged from 49.2% to 76.3%, and the mean NIHSS score at admission ranged from 2.4 to 16.0 when reported. Three studies exclusively enrolled patients undergoing EVT (21, 24, 26), whereas the remaining studies (14, 16–20, 22, 23, 25, 27) included general AIS populations. MES were detected using TCD in all studies, with bilateral middle cerebral artery monitoring performed universally. The timing of MES assessment varied from admission, within 6–72 h of stroke onset or admission, to immediately after EVT. Monitoring durations ranged from 30 to 60 min. Accordingly, 361 patients (20.3%) had detectable MES. Follow-up durations varied, including discharge outcomes (16, 18, 22), 14-day (17, 19, 20, 27), 90-day (14, 21, 23, 24, 26), and 180-day assessments (25). The number of patients with poor functional outcomes ranged from 18 to 96. Seven studies (14, 16–18, 22, 26, 27) adjusted for potential confounding variables, commonly including age, sex, baseline stroke severity, vascular risk factors, prior functional status, imaging findings, and treatment-related variables, to a varying degree, whereas six studies (19–21, 23–25) reported unadjusted analyses only.

**Table 1:** Characteristics of the included studies.

Study	Country	Design	No. of patients with AIS	Mean age (years)	Men (%)	Mean NIHSS at admission	EVT (%)	Timing of MES assessment	Protocol of MES evaluation	No. of patients with MES	Follow-up duration	No. of patients with poor functional outcome	Variables adjusted
Seren 2000	Spain	PC	182	65.1	74.2	NR	NR	At admission	Bilateral TCD, both MCAs, 30 min monitoring	17	Until discharge	48	Age, stroke severity on admission (in CSS), presence of arterial or cardiac embolic source
Idicula 2010	Norway	PC	40	70	72.5	7	NR	Within 6 h of stroke onset	Bilateral TCD, both MCAs, 60 min monitoring	10	Until discharge	NR	Age, sex, NIHSS at admission
Jiang 2012	China	PC	135	65	76.3	4.7	NR	Within 48 h of stroke onset	Bilateral TCD, both MCAs, 30 min monitoring	28	90 days	19	None
Li 2017	China	PC	238	66.8	68.1	NR	NR	Within 24 h of admission	Bilateral TCD, both MCAs, 30 min monitoring	50	14 days	62	Age, sex, smoking, drinking, hypertension, DM, prior stroke, CAD, hyperlipidemia, SUA, admission NIHSS, and hospital stay
Farina 2018	Italy	PC	40	65.8	57.5	16	100	Immediately after EVT	Bilateral TCD, both MCAs, 60 min monitoring	26	90 days	20	None
Ping 2020	China	PC	240	64.2	50.4	NR	NR	Within 24 h of admission	Bilateral TCD, both MCAs, 40 min monitoring	35	14 days	51	None
Das 2020	USA	RC	248	62.6	62.9	3.6	11.7	Within 48 h of admission	Bilateral TCD, both MCAs MCA (primarily), 60 min monitoring	38	Until discharge	96	Age, sex, admission NIHSS, baseline mRS, carotid stenosis, EVT, ischemic recurrence, length of stay
Sheriff 2020	USA	PC	111	70	51.4	15	100	Within 72 h of stroke onset	Bilateral TCD, both MCAs (M1 segment), 30 min monitoring	43	90 days	52	Age, NIHSS, LSW-to-recanalization time, ASPECTS, mTICI grade, smoking, NIHSS at admission, cholesterol, TOAST
Bazan 2020	Brazil	PC	111	68	63.1	NR	NR	Within 48 h of stroke onset	Bilateral TCD, both MCAs, 30–45 min monitoring	8	180 days	44	None
Zhu 2021	China	RC	83	67	67.5	3.6	NR	Within 24 h of admission	Bilateral TCD, both MCAs, 30 min monitoring	19	14 days	26	None
Wan 2021	China	RC	212	76.3	73.6	2.4	NR	Within 24 h of admission	Bilateral TCD, both MCAs, 60 min monitoring	56	14 days	90	Age, sex, blood lipids, CRP, BUN, Scr, BNP, and Lp-PLA2
Castro 2024	Portugal, Croatia, Italy, Czech Republic, and Germany	PC	61	78	49.2	11	42.6	Within 24 h of stroke onset	Bilateral TCD, both MCAs (M1 segment), 60 min monitoring	14	90 days	31	Age, sex, BNP, antiplatelets, NIHSS at admission, thrombectomy, anterior circulation, previous mRS, and stenosis
Wan 2025	China	PC	76	58.2	55.3	NR	100	Immediately after EVT	Bilateral TCD, both MCAs, 30 min monitoring	17	90 days	18	None

AIS, acute ischemic stroke; ASPECTS, Alberta Stroke Program Early CT Score; BNP, B-type natriuretic peptide; BUN, blood urea nitrogen; CAD, coronary artery disease; CRP, C-reactive protein; CSS, Canadian Stroke Scale; DM, diabetes mellitus; EVT, endovascular treatment; Lp-PLA2, lipoprotein-associated phospholipase A2; LSW, last-seen-well; M1, first segment of the middle cerebral artery; MCA, middle cerebral artery; MES, microembolic signals; mRS, modified Rankin Scale; mTICI, modified Thrombolysis in Cerebral Infarction; NIHSS, National Institutes of Health Stroke Scale; NR, not reported; PC, prospective cohort; RC, retrospective cohort; Scr, serum creatinine; SUA, serum uric acid; TCD, transcranial Doppler ultrasonography; TOAST, Trial of Org 10172 in Acute Stroke Treatment classification.

### Study quality evaluation

The methodological quality of the included studies was assessed using the NOS, and the results are presented in Table 2. NOS scores ranged from 6 to 9 points, with a median score of 7. Two studies (14, 26) achieved the maximum score of 9, reflecting excellent methodological quality with representative cohorts, adequate adjustment for important confounding variables, rigorous outcome assessment, and sufficient follow-up. Three studies (16, 17, 22) scored 8, indicating generally high methodological quality with only minor limitations. Six studies scored 7 (18, 21, 23–25, 27), while the remaining two studies (19, 20) scored 6, primarily because of limited adjustment for confounding factors and relatively short follow-up durations. Overall, ascertainment of exposure and outcome assessment was adequate across all studies, and all cohorts were confirmed to be free of the outcome of interest at baseline. Most studies used standardized TCD protocols for MES detection and clearly defined functional outcomes. Although adjustment strategies varied considerably, seven studies (14, 16–18, 22, 26, 27) controlled for key prognostic factors such as age, sex, baseline NIHSS score, vascular risk factors, and treatment-related variables. Despite some concerns regarding cohort representativeness, limited confounder adjustment, and short follow-up duration in a subset of studies, the overall methodological quality was considered moderate to high, supporting the reliability of the pooled estimates. Nevertheless, variations in patient populations, timing of MES assessment, monitoring protocols, follow-up duration, and analytical models may have contributed to residual heterogeneity among the included studies.

**Table 2:** Study quality evaluation via the Newcastle-Ottawa Scale.

Study	Representativeness of the exposed cohort	Selection of the non-exposed cohort	Ascertainment of exposure	Outcome not present at baseline	Control for age and sex	Control for other confounding factors	Assessment of outcome	Enough long follow-up duration	Adequacy of follow-up of cohorts	Total
Seren 2000	1	1	1	1	1	1	1	0	1	8
Idicula 2010	1	1	1	1	1	1	1	0	1	8
Jiang 2012	1	1	1	1	0	0	1	1	1	7
Li 2017	1	1	1	1	1	1	1	0	1	8
Farina 2018	1	1	1	1	0	0	1	1	1	7
Ping 2020	1	1	1	1	0	0	1	0	1	6
Das 2020	0	1	1	1	1	1	1	0	1	7
Sheriff 2020	1	1	1	1	1	1	1	1	1	9
Bazan 2020	1	1	1	1	0	0	1	1	1	7
Zhu 2021	1	1	1	1	0	0	1	0	1	6
Wan 2021	0	1	1	1	1	1	1	0	1	7
Castro 2024	1	1	1	1	1	1	1	1	1	9
Wan 2025	1	1	1	1	0	0	1	1	1	7

### Association between MES and functional outcome of patients with AIS

**Figure 2:**
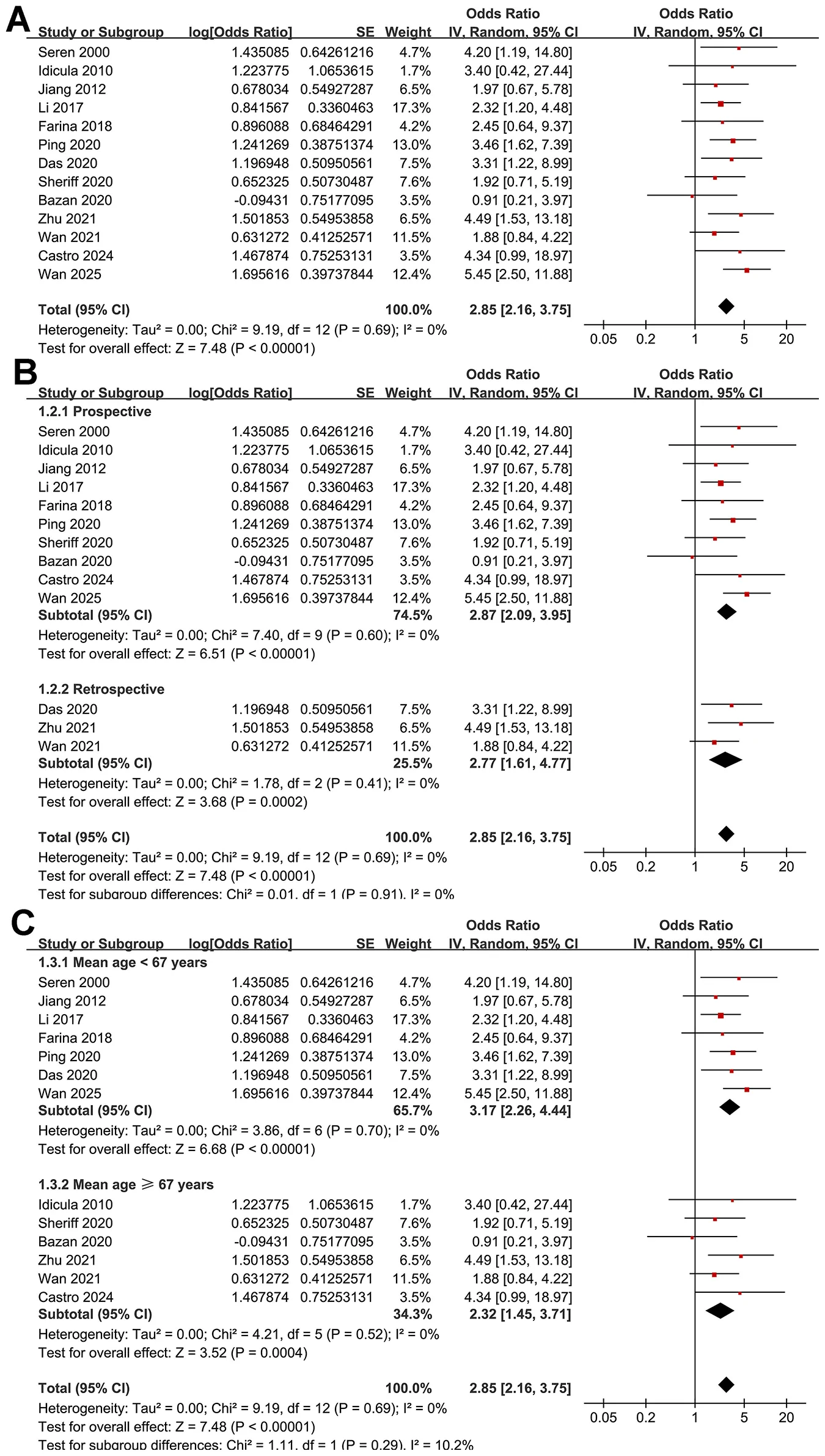
Forest plots showing the meta-analysis of the association between MES and poor functional outcome of patients with AIS: **(A)** forest plots for the overall meta-analysis; **(B)** forest plots for the subgroup analysis according to study design; and **(C)** forest plots for the subgroup analysis according to the mean ages of the patients.

**Figure 3:**
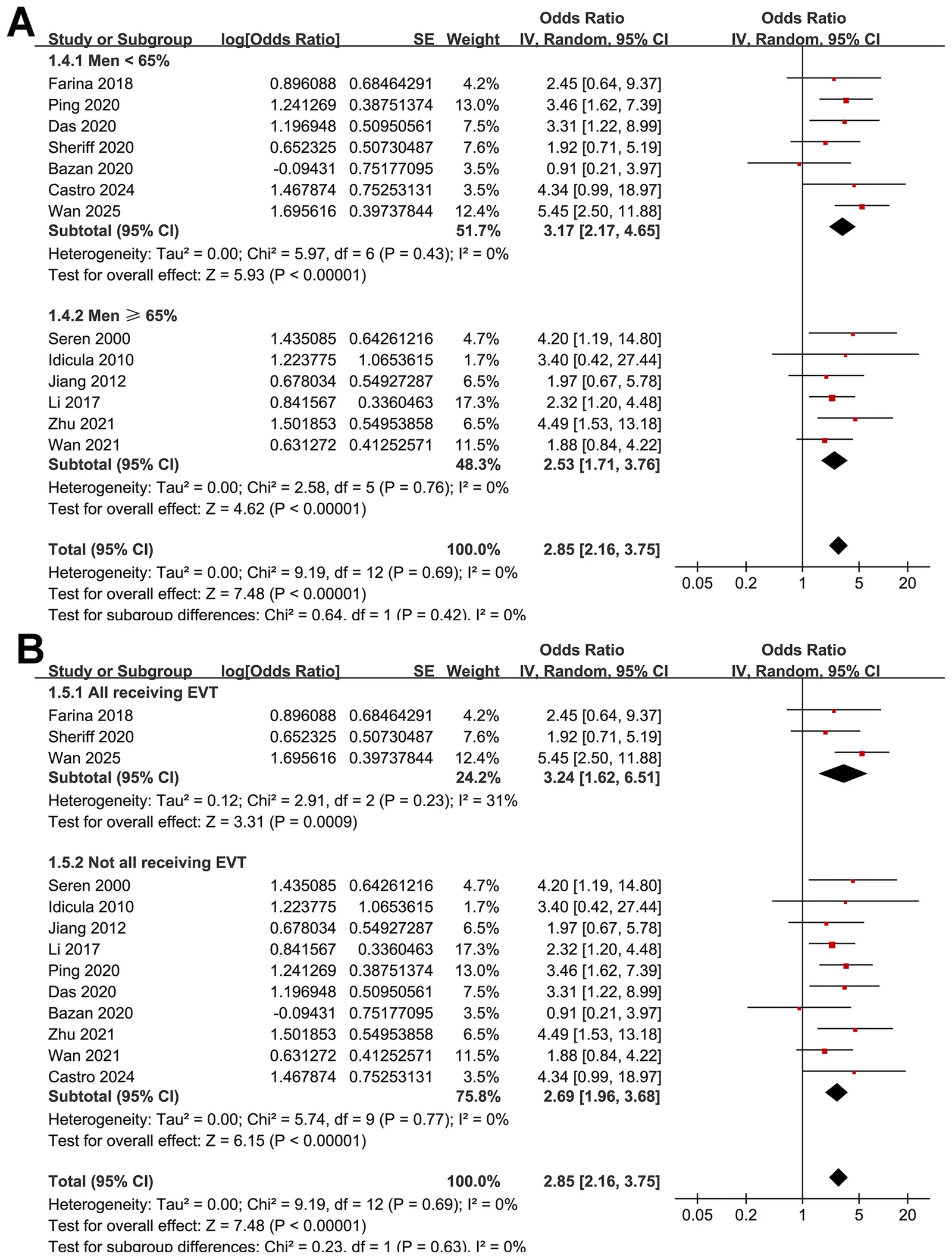
Forest plots showing the meta-analysis of the association between MES and poor functional outcome of patients with AIS: **(A)** forest plots for the subgroup analysis according to the proportion of men; and **(B)** forest plots for the subgroup analysis according to the EVT status.

**Figure 4:**
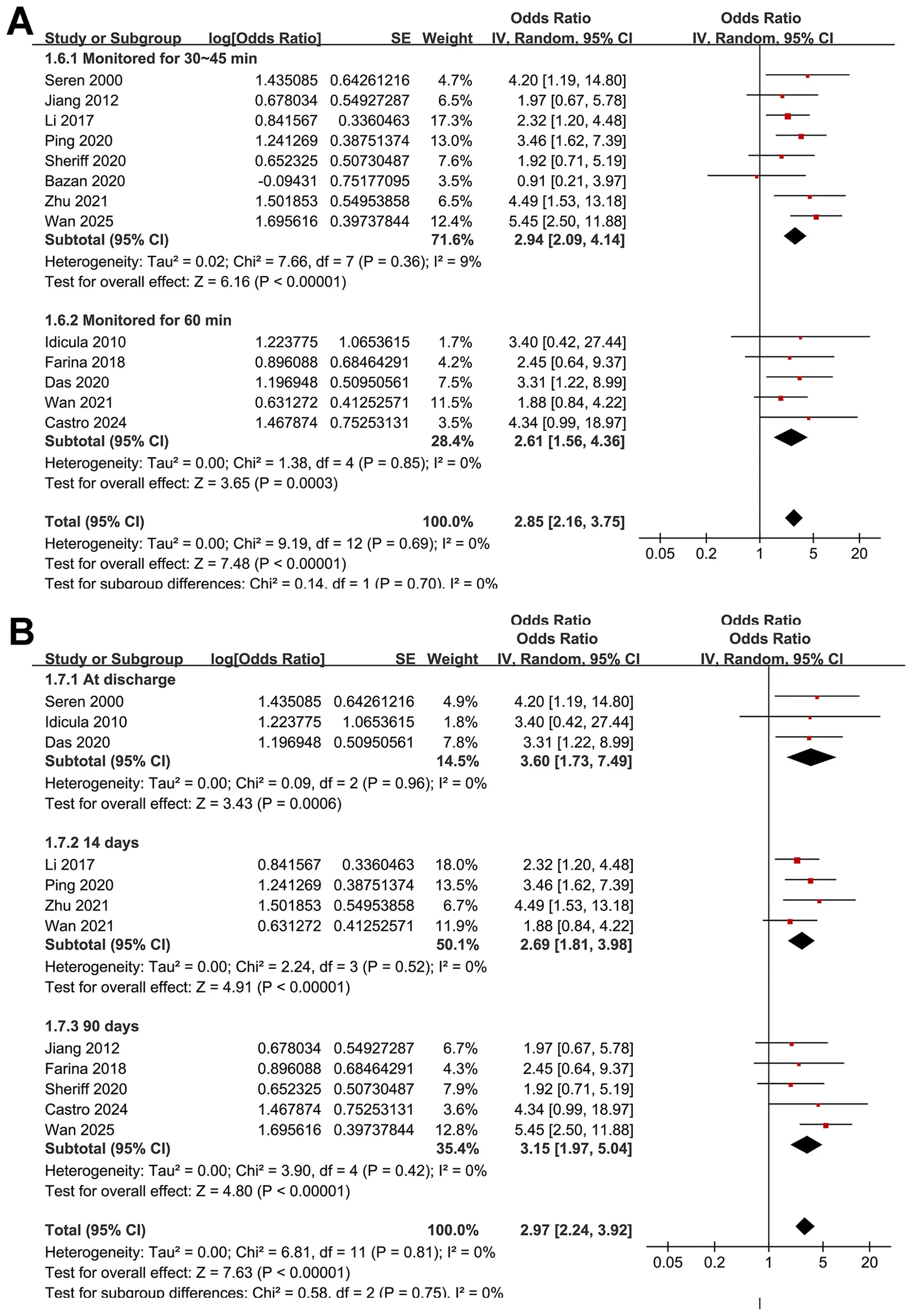
Forest plots showing the meta-analysis of the association between MES and poor functional outcome of patients with AIS: **(A)** forest plots for the subgroup analysis according to the duration of TCD monitoring for MES detection; and **(B)** forest plots for the subgroup analysis according to the follow-up durations.

**Figure 5:**
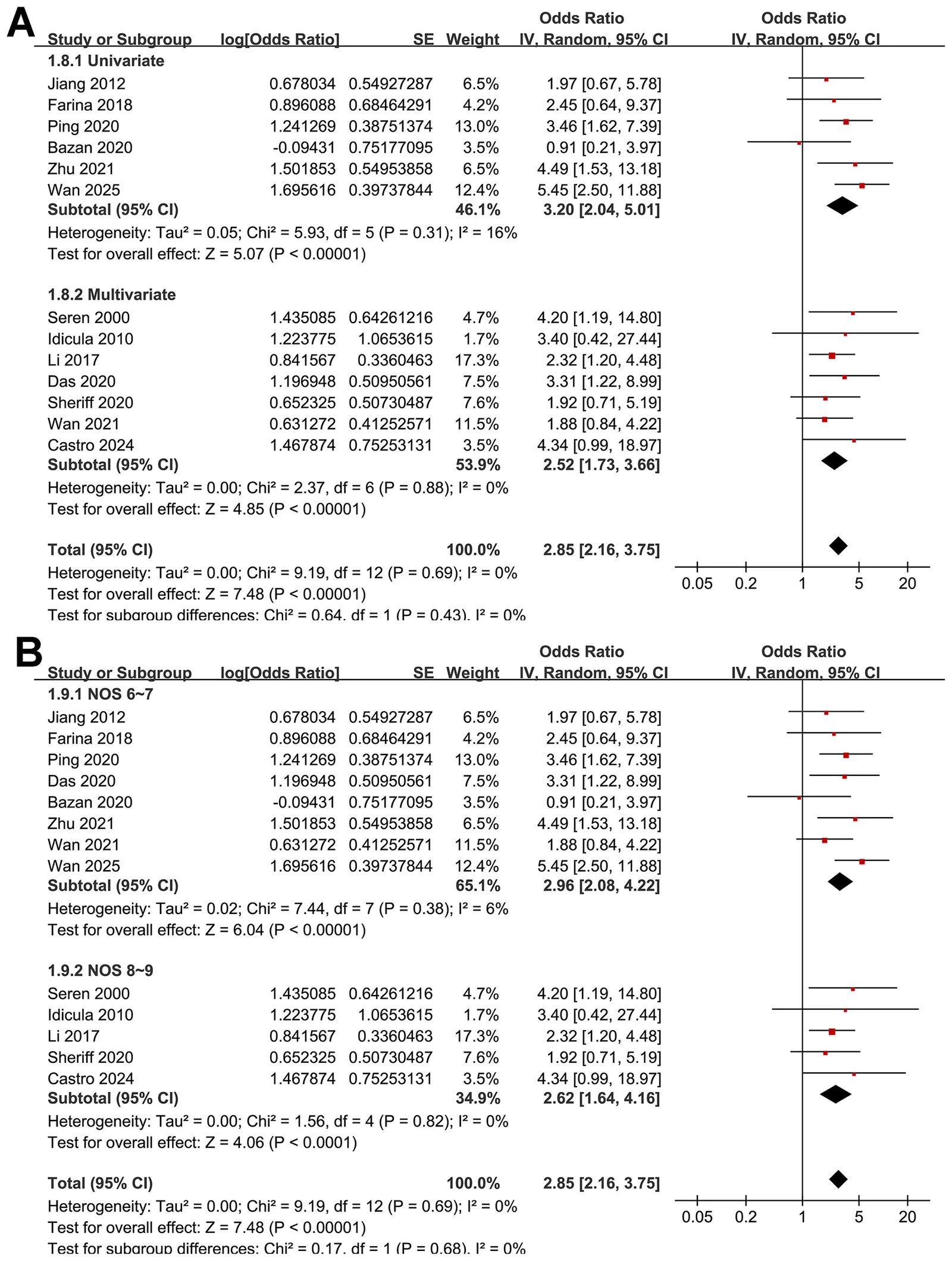
Forest plots showing the meta-analysis of the association between MES and poor functional outcome of patients with AIS: **(A)** forest plots for the subgroup analysis according to the analytical model; and **(B)** forest plots for the subgroup analysis according to the study quality score.

The pooled analysis of the 13 studies (14, 16–27) showed that MES detected on TCD after AIS was associated with an increased risk of poor functional outcome during follow-up (OR: 2.85, 95% CI: 2.16 to 3.75, *p* < 0.001; Figure 2A) without significant heterogeneity (*p* for Cochrane Q test = 0.69; *I*^2^ = 0%). Leave-one-out sensitivity analyses showed similar results, with OR ranging from 2.60 to 3.00 (all *p* < 0.05), indicating the robustness of the overall estimate. Further subgroup analyses showed consistent results in prospective and retrospective cohorts (OR: 2.87 vs. 2.77, *p* for subgroup difference = 0.91; Figure 2B), in patients with the mean age < 67 years and ≥ 67 years (OR: 3.17 vs. 2.32, *p* for subgroup difference = 0.29; Figure 2C), and between patients with the proportion of men < 65% and ≥ 65% (OR: 3.17 vs. 2.53, *p* for subgroup difference = 0.42; Figure 3A). The results were not statistically significant between studies including patients receiving EVT only and studies not restricted to EVT-treated patients (OR: 3.24 vs. 2.69, *p* for subgroup difference = 0.63; Figure 3B). In addition, consistent results were observed in studies with the duration of TCD monitoring for MES detection of 30–45 min and 60 min (OR: 2.94 vs. 2.61, *p* for subgroup difference = 0.70; Figure 4A). Further subgroup analyses according to follow-up duration showed that MES were associated with an increased risk of poor functional outcome at discharge, 14 days, and 90 days (OR: 3.60, 2.69, and 3.15, respectively, *p* for subgroup difference = 0.75; Figure 4B). Only subgroups represented by at least three studies were included in this analysis. Finally, similar results were obtained for studies with univariate and multivariate analyses (OR: 3.20 vs. 2.52, *p* for subgroup difference = 0.43; Figure 5A), and in studies with the NOS scores of 6–7 and 8–9 (OR: 2.98 vs. 2.62, *p* for subgroup difference = 0.68; Figure 5B).

### Publication bias

**Figure 6:**
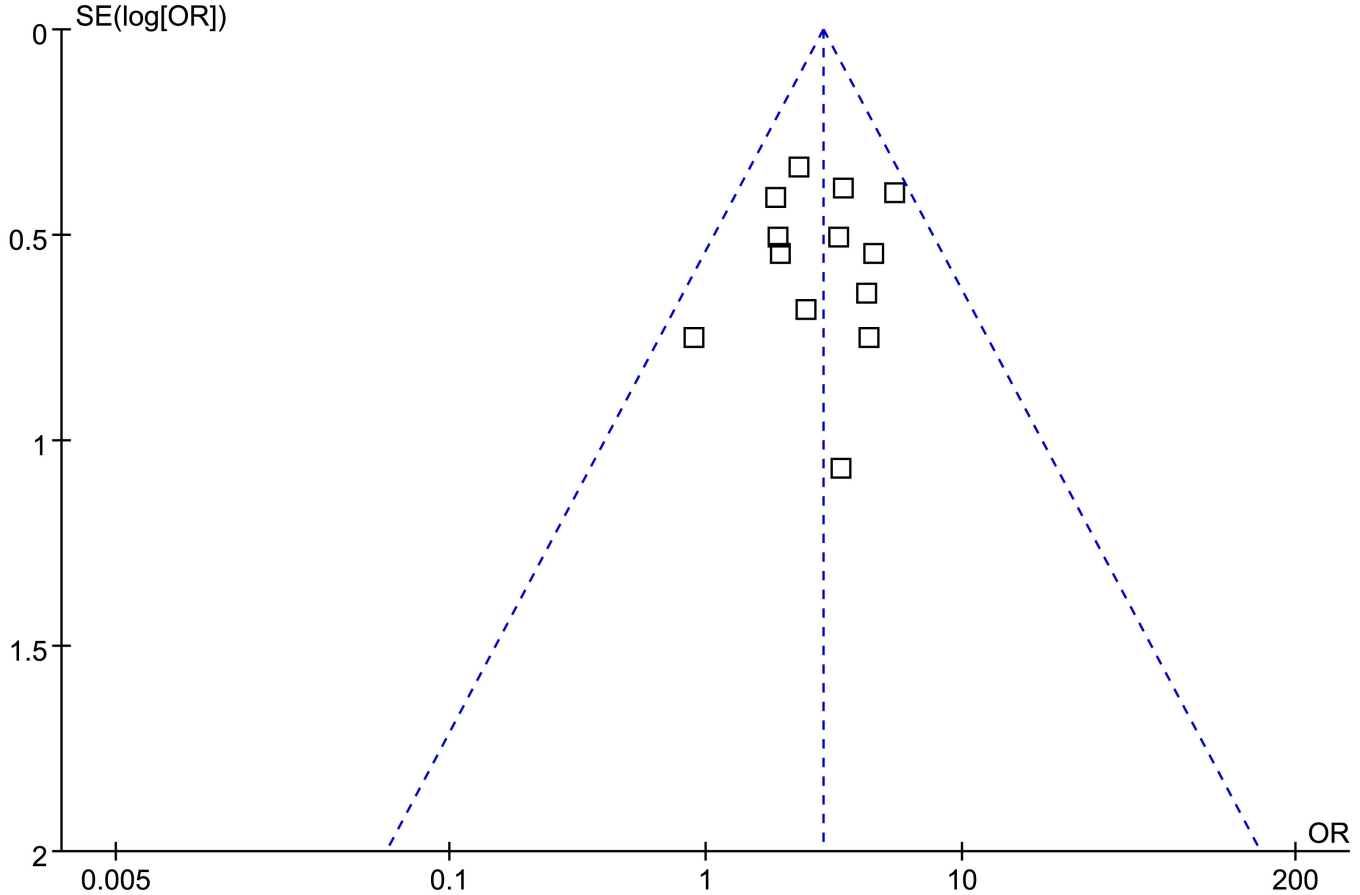
Funnel plots evaluating the publication bias for the meta-analysis of the association between MES and poor functional outcome of patients with AIS.

Assessment of publication bias suggested no obvious evidence of reporting-related distortions. Funnel plots for the meta-analysis (Figure 6) appeared largely symmetrical, and Egger's regression test was non-significant (*p* = 0.72), indicating no detectable small-study effects. However, these findings should be interpreted cautiously because only 13 studies contributed to the meta-analysis, limiting the statistical power of publication bias assessments.

## Discussion

In this meta-analysis of 13 cohort studies involving 1,777 patients with AIS, MES detected by TCD were associated with an increased risk of poor functional outcome. The association remained robust across sensitivity and subgroup analyses, with no significant effect modification by study design, demographic characteristics, EVT status, TCD monitoring duration, analytical model, or study quality. Although statistical heterogeneity was negligible (*I*^2^ = 0%), clinical and methodological differences remained across studies, particularly in stroke characteristics, MES monitoring protocols, and outcome assessment. These findings support MES as a potentially relevant prognostic marker after AIS.

Several pathophysiological and clinical mechanisms may explain the observed association between MES and poor functional outcomes. MES are generally considered markers of ongoing cerebral embolization and may reflect an active embolic source, such as unstable atherosclerotic plaques, intraluminal thrombi, or cardioembolic lesions (13, 36, 37). Their association with poor functional outcome may therefore reflect persistent thromboembolic activity and recurrent cerebral ischemic insults (28, 38, 39). However, an alternative and not mutually exclusive explanation is that MES serve as a surrogate marker of greater disease severity or more unstable underlying vascular or cardiac pathology rather than directly contributing to poor recovery. Thus, although recurrent microembolization could plausibly contribute to infarct expansion, impaired collateral circulation, or additional ischemic lesions, the present observational evidence cannot establish such a causal pathway (40).

In addition, MES appear to be closely linked to disease severity and ongoing cerebrovascular instability. Hao et al. reported that MES were associated with a greater number of ischemic lesions and a higher prevalence of multiple infarcts on diffusion-weighted imaging in patients with acute stroke and large artery occlusive disease (41). Moreover, persistence of MES during the acute phase was associated with less neurological improvement, suggesting that MES may reflect sustained pathological activity that interferes with neurological recovery (41). Similarly, Sun et al. found that patients with positive MES were less likely to demonstrate improvement in NIHSS scores during the first week after stroke, further supporting the relationship between MES and unfavorable neurological evolution (42). These observations are clinically relevant because early neurological recovery is one of the strongest determinants of long-term functional independence after AIS (43). Therefore, MES may serve as an indirect marker of persistent ischemic injury and impaired neurological restitution. The prognostic significance of MES may also extend beyond functional disability. Ferreira et al. recently reported worse one-year cognitive outcomes among MES-positive patients independent of conventional prognostic factors, suggesting that persistent microembolization may reflect a broader cerebrovascular disease burden affecting multiple domains of post-stroke recovery (44).

The subgroup analyses provide further support for the robustness of the findings. The association between MES and poor functional outcome was observed across prospective and retrospective studies and remained significant regardless of patient age, sex distribution, TCD monitoring duration, EVT status, analytical model, and study quality. Follow-up duration warrants particular consideration because disability at discharge or within the first weeks after AIS predominantly reflects the initial neurological injury and early complications, whereas 90-day outcomes additionally incorporate subsequent neurological recovery, rehabilitation, and post-stroke events. Despite these clinical differences, MES were associated with poor functional outcome at discharge, 14 days, and 90 days, suggesting that the observed association was not confined to early disability. Nevertheless, the limited number of studies at each timepoint precludes firm conclusions regarding whether the association changes over the course of recovery. More broadly, the absence of significant subgroup differences suggests that the association may be relatively consistent across the evaluated study characteristics. However, these findings do not establish MES as an independent prognostic factor, particularly given the limited number of studies within some subgroups and variability in confounder adjustment. The consistent findings from sensitivity analyses further support the stability of the pooled estimate.

Several strengths of this study should be acknowledged. First, to our knowledge, this represents the most up-to-date meta-analysis specifically evaluating the association between MES and functional outcomes in patients with AIS. Second, only cohort studies were included, thereby strengthening the temporal relationship between MES detection and subsequent outcomes. Third, the methodological quality of the included studies was generally moderate to high. Fourth, extensive subgroup and sensitivity analyses consistently demonstrated robust findings without evidence of substantial statistical heterogeneity, supporting the stability of the pooled association.

Nevertheless, several limitations should be considered when interpreting the results. First, although most studies were prospective, several retrospective studies were included, which may be susceptible to selection and information bias (45). Second, publication bias cannot be completely excluded because smaller studies with null findings may have remained unpublished. Third, substantial clinical heterogeneity existed among studies regarding stroke severity, demographic characteristics, stroke etiology, reperfusion treatments, timing of TCD monitoring, monitored vessels, monitoring duration, and follow-up schedules. Among these factors, stroke etiology is particularly relevant because the prevalence and potential source of MES may differ substantially among large-artery atherosclerosis, cardioembolism, small-vessel disease, and cryptogenic or undetermined stroke (28). Although several included studies reported etiologic classifications, they did not provide etiology-specific effect estimates for the association between MES and functional outcome (16, 22, 25). Therefore, a reliable subgroup analysis according to stroke etiology could not be performed. Future studies should report these associations separately across well-defined stroke etiologies. Although statistical heterogeneity was low, the influence of these factors could not be comprehensively evaluated because individual patient data were unavailable. Fourth, both adjusted and unadjusted effect estimates were included, and adjustment strategies varied considerably across studies. Although subgroup analysis showed similar associations for univariate and multivariate estimates, combining these estimates may still introduce residual confounding. Moreover, important determinants of functional outcome, including baseline stroke severity, infarct volume, recanalization status, premorbid disability, antithrombotic therapy, and plaque characteristics, were not consistently accounted for across studies. Therefore, the independent prognostic value of MES cannot be firmly established. Fifth, because all included studies were observational, causality cannot be inferred. MES may represent a surrogate marker of greater stroke severity, persistent embolic activity, or unstable underlying vascular pathology rather than a direct mediator of poor functional outcome. Finally, only studies published in English or Chinese were included, which may have introduced language bias.

Despite these limitations, the present findings have important clinical implications. TCD is a noninvasive, widely available, repeatable, and relatively inexpensive technique that can be performed at the bedside without radiation exposure. In addition to MES, other TCD-derived parameters, including mean flow velocity (MFV) and pulsatility index (PI), have been investigated as potential prognostic markers after AIS (46). A recent systematic review and meta-analysis showed that an elevated MFV index was associated with an increased risk of poor functional recovery at 90 days after successful mechanical thrombectomy, although the prognostic value of other parameters, including PI, was less consistent (46). These findings, together with our results, suggest that TCD may provide multidimensional prognostic information after AIS, with MES reflecting ongoing embolic activity and hemodynamic parameters providing complementary information on cerebral perfusion. Identification of MES during the acute phase of AIS may help identify patients at increased risk of poor functional outcome and potentially inform closer clinical monitoring (47). However, whether MES add clinically meaningful predictive value beyond established prognostic factors remains uncertain. The available studies primarily evaluated associations after adjustment for selected covariates and did not consistently compare prediction models with and without MES. Future prospective studies should therefore evaluate the incremental value of MES beyond established clinical and imaging predictors using measures of discrimination, calibration, and risk reclassification. Such studies should also standardize TCD monitoring protocols, evaluate MES burden and persistence, investigate interactions with reperfusion and antithrombotic therapies, and determine whether interventions targeting persistent embolization improve clinical outcomes.

## Conclusions

In conclusion, this meta-analysis demonstrates that MES detected by TCD are associated with an increased risk of poor functional outcomes in patients with AIS. However, whether MES provide independent or incremental prognostic value beyond established clinical and imaging predictors remains uncertain. Given the observational nature of the available evidence and the possibility of residual confounding, these findings should be confirmed in future well-designed prospective studies.

## Data Availability

The original contributions presented in the study are included in the article/Supplementary material, further inquiries can be directed to the corresponding author.
