# Supplementary material for: Microembolic signals in transcranial Doppler and the functional outcome of patients with acute ischemic stroke: a meta-analysis
Source: Front Neurol. 2026 Sep 10;17:1911457. doi: 10.3389/fneur.2026.1911457 (PMC13600997; doi:10.3389/fneur.2026.1911457)
Supplement: Supplementary file 1 [file Table_1.docx]

**Detailed search strategy for each database**

**PubMed**

(("Stroke"[Mesh] OR stroke* OR "ischemic stroke" OR "ischaemic stroke" OR "cerebral infarction" OR "brain infarction" OR "acute ischemic stroke" OR AIS) AND ("Embolism"[Mesh] OR "microembolic signal*" OR "microembol*" OR MES OR "high-intensity transient signal*" OR HITS) AND ("Transcranial Doppler Ultrasonography"[Mesh] OR "transcranial Doppler" OR TCD OR "transcranial color-coded Doppler" OR TCCD) AND ("modified Rankin Scale" OR mRS OR "functional outcome" OR prognosis OR outcome* OR disability OR dependency))

**Embase**

('stroke'/exp OR stroke*:ti,ab,kw OR 'ischemic stroke':ti,ab,kw OR 'cerebral infarction':ti,ab,kw) AND ('microembolism'/exp OR 'microembolic signal*':ti,ab,kw OR microembol*:ti,ab,kw OR MES:ti,ab,kw OR HITS:ti,ab,kw) AND ('transcranial doppler ultrasonography'/exp OR 'transcranial doppler':ti,ab,kw OR TCD:ti,ab,kw OR TCCD:ti,ab,kw) AND ('modified rankin scale':ti,ab,kw OR mRS:ti,ab,kw OR prognosis:ti,ab,kw OR outcome*:ti,ab,kw OR disability:ti,ab,kw)

**Web of Science**

TS=((stroke* OR "ischemic stroke" OR "ischaemic stroke" OR "cerebral infarction") AND ("microembolic signal*" OR microembol* OR MES OR HITS) AND ("transcranial doppler" OR TCD OR TCCD) AND ("modified Rankin Scale" OR mRS OR prognosis OR outcome* OR disability)))

**Wanfang**

(微栓子信号 OR 微栓子 OR 栓子信号 OR 微栓塞信号 OR MES OR HITS) AND (经颅多普勒 OR TCD) AND (脑梗死 OR 缺血性卒中) AND (预后 OR 转归 OR 功能结局 OR mRS OR Rankin)

**CNKI**

(微栓子信号 OR 微栓子 OR 栓子信号 OR 微栓塞信号 OR MES OR HITS) AND (经颅多普勒 OR TCD OR 经颅多普勒超声) AND (脑梗死 OR 缺血性卒中 OR 缺血性脑卒中 OR 急性脑梗死) AND (预后 OR 转归 OR 功能结局 OR 改良Rankin量表 OR mRS)
